# Canine dermatophytosis due to *Arthroderma uncinatum*: case report and diagnostic considerations

**DOI:** 10.1016/j.mmcr.2026.100812

**Published:** 2026-07-17

**Authors:** Rossella Samarelli, Domenico Otranto, Mara Miglianti, Marcos Antonio Bezerra-Santos, Gustavo Giusiano

**Affiliations:** a Department of Veterinary Medicine, University of Bari “Aldo Moro”, Str. prov. per Casamassima km 3, Valenzano, Bari, 70010, Italy; b Department of Veterinary Clinical Sciences, Jockey Club College of Veterinary Medicine and Life Sciences, City University of Hong Kong, Kowloon, Hong Kong, China; c Faculty of Veterinary Science, Chulalongkorn University in, Bangkok, Thailand; d Mycology Department, Institute of Regional Medicine, Northeast National University, CONICET, Resistencia, 3500, Argentina

**Keywords:** Canine dermatophytosis, Geophilic, *Arthroderma uncinatum*, Atypical presentation, Sequencing, One health

## Abstract

This report describes a molecularly confirmed case of canine dermatophytosis caused by *Arthroderma uncinatum* in an 11-month-old English Setter from Southern Italy. The dog presented with two alopecic, erythematous, scaling lesions on the metacarpal and metatarsal regions. While routine microscopic examinations were inconclusive, dermatophyte culture yielded a fungal isolate identified as *A. uncinatum* by ITS sequencing. Topical 1% clotrimazole administered for three weeks resulted in complete clinical and mycological cure. This case documents an uncommon infection by a geophilic dermatophyte and expands veterinary knowledge of *A. uncinatum*-associated canine disease.

## 1 Introduction

Canine dermatophytosis is a superficial fungal infection of keratinized tissues in companion animals, colonizing the stratum corneum, hair shafts, and claws [1]. Caused by a specialized group of filamentous keratinophilic fungi, the disease in dogs is predominantly attributed to the zoophilic species *Microsporum canis*, followed by members of the *Trichophyton mentagrophytes* complex [2,3]. Although dermatophytosis is generally regarded as self-limiting in immunocompetent hosts, its clinical manifestation is highly heterogeneous, ranging from focal alopecic, erythematous, and scaling lesions to inflammatory, nodular, ulcerative, or pyogranulomatous presentations, such as kerions. This marked pleomorphism, coupled with atypical anatomical localizations, including the distal extremities and interdigital regions, makes its clinical recognition challenging. Unusual presentations can also mimic different conditions such as superficial bacterial pyoderma, demodicosis, allergic dermatitis, foreign-body reactions, traumatic injuries, and sterile inflammatory or immune-mediated disorders [4]. This extensive range of symptoms not only misleads clinical recognition but also risks delaying an accurate diagnosis and appropriate therapeutic intervention [5].

Diagnosis becomes significantly more challenging when infections are caused by geophilic dermatophytes. These fungi are primarily associated with soil and other environmental sources. Infection occurs through contact with contaminated substrates rather than direct transmission from an infected host. The sporadic occurrence of these infections, the lack of a clear epidemiological link, and their ability to induce a marked inflammatory response that mimics other dermatological conditions can complicate clinical suspicion [6,7]. Although naturally occurring infections are uncommon, these fungi can cross the saprophytic-pathogenic divide and induce clinically relevant disease after environmental exposure, particularly when cutaneous barriers are disrupted or via repeated contact with contaminated fomites. In dogs, exposure to geophilic dermatophytes mainly occurs in outdoor living animals and in geographic regions characterized by warm climates, such as those in the Mediterranean basin, including Southern Italy [2]. When these opportunistic soil fungi breach the cutaneous barrier of a susceptible animal, they carry substantial public health implications. Under the "One Health" framework, companion animals sharing domestic environments serve as critical reservoirs for human dermatophytosis; exposure to contaminated hair, dander, or soil-laden fomites regularly triggers outbreaks among pet owners, veterinary personnel, and shelter workers [8]. While anthropozoonotic transmission is historically dominated by *M. canis*, the epidemiological role of atypical geophilic dermatophytes is increasingly investigated [9]. Human contact with these opportunistic agents typically occurs through shared microhabitats containing keratinous debris, where the fungi induce atypical inflammatory reactions that defy conventional diagnostic algorithms designed primarily for classic zoophilic phenotypes.

Within the shifting taxonomy of these soil saprophytes, *Arthroderma uncinatum* (formerly *Trichophyton ajelloi* under the anamorph-teleomorph nomenclature), represents a rare opportunistic pathogen in both veterinary and human medicine [10,11]. Despite its wide ecological distribution and marked keratinolytic activity in soil, *T. ajelloi* has traditionally been considered to be of low pathogenic potential, being isolated so far more from environmental samples than from clinical lesions [9]. Reports of clinically significant infections remain exceptional, with *T. ajelloi* only sporadically implicated in superficial dermatophytosis, inducing localized cutaneous lesions following traumatic lesions, thus direct environmental exposure [7,12]. Evidence in veterinary medicine is even more limited as *A. uncinatum* has been documented mainly as a transient, non-pathogenic component of the fungal biota recovered from the hair coats of healthy dogs [13].

In recent years, molecular characterization based on sequencing of the internal transcribed spacer (ITS) region has improved species-level identification, partially overcoming limitations inherent to the morphology-based classification for uncommon, polymorphic, or phylogenetically related taxa [9,14]. To date, molecularly confirmed canine dermatophytosis associated with active lesions caused by *A. uncinatum* remains undocumented in the indexed veterinary literature. In this case report, we describe an atypical case of dermatological lesions involving the metacarpal and metatarsal regions of a dog from Apulia (Southern Italy), molecularly identified as *A. uncinatum*.

## 2 Case presentation

**Fig. 1:**
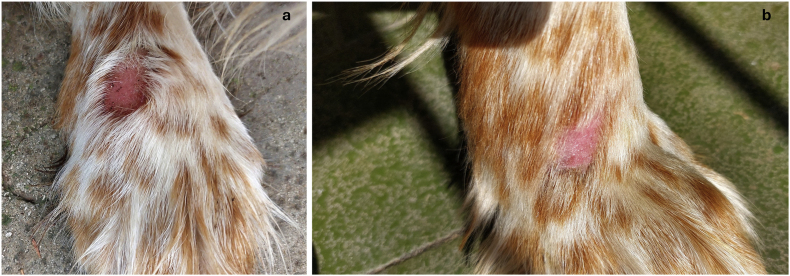
Clinical presentation of atypical dermatophytosis lesions caused by *Arthroderma uncinatum*: (**a**) metacarpal region of the right forelimb; (**b**) metatarsal region of the right hindlimb.

An 11-month-old, intact male English Setter was presented to the Parasitic Diseases Unit of the Department of Veterinary Medicine, University of Bari “Aldo Moro” (Apulia, Italy), for the evaluation of cutaneous lesions affecting the metacarpal and metatarsal regions (Day 0). General physical examination revealed no abnormalities. Dermatological examination identified two round, alopecic lesions located on two distinct limbs: one on the metacarpal region of the right forelimb and the other on the metatarsal region of the right hindlimb. The alopecic skin appeared mildly (hind leg) to moderately (fore leg) erythematous, with fine scaling on its periphery (Fig. 1a–b). Pruritus and pain upon palpation were not evident. The patient was up to date with vaccinations and, one month prior to presentation, had received a canine leishmaniosis vaccine. The dog was kept exclusively outdoors, with restricted garden access. The patient cohabited with another 12-year-old English Setter that shared the same environment. Both dogs were undergoing outdoor field training for hunting, involving frequent and prolonged exposure to soil, vegetation, and diverse environmental substrates. The cohabitant dog did not show any similar dermatological lesions. The initial differential diagnosis included dermatophytosis, superficial bacterial pyoderma and demodicosis.

### 2.1 Sample collection and direct examination

At the time of the clinical assessment (Day 0), the patient underwent tape-strip cytology, a trichogram, a skin scraping, and a dermatophyte culture. Samples were collected using a tape strip and stained using a Diff-Quick stain. Microscopic findings at 100x magnification showed only sporadic extracellular coccoid bacteria. Plucked hairs collected from the margins of the lesions were examined at 10x magnification and showed a good proportion between the anagen and telogen phases, a normal hair shaft shape, and an absence of ectoparasites and fungal elements. Skin scrapings were collected using a no. 10 scalpel blade. The affected cutaneous areas were cleansed with 70% isopropyl alcohol to minimize the surface bacterial contamination. Hair shafts, skin scrapings, and scales were collected from the margins of the lesions. For direct examination, a portion of the samples were placed on glass slides, suspended in a 10% potassium hydroxide (KOH) solution, and gently heated to accelerate tissue clearing. Under a light microscope at 40× magnification, no fungal structures or ectoparasites were observed. Given the lesions’ clinical appearance, a dermatophyte culture was performed on Sabouraud Dextrose Agar (SDA) supplemented with chloramphenicol (0.05 mg/mL) [Liofilchem®, Italy] and on Mycobios selective agar (MYC) (Biolife®, Milan, Italy). Cultures were incubated aerobically at 25°C and inspected daily for 21 days. Considering the clinical appearance of the lesions compatible with dermatophytosis, a new sample was taken after 48 hours (Day +2).

### 2.2 Fungal isolation and phenotypic characterization

**Fig. 2:**
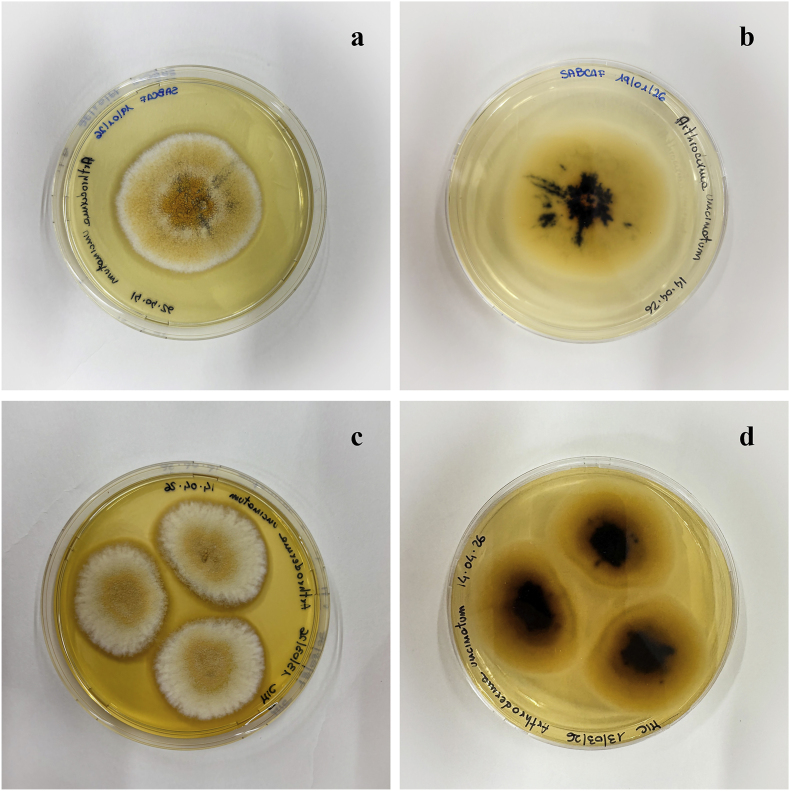
Macroscopic colonial morphology of *Arthroderma uncinatum* on Sabouraud Dextrose Agar (SDA) and Mycobios Selective Agar (MYC) at day 21: (**a-c**) obverse view; (**b-d**) reverse view.

**Fig. 3:**
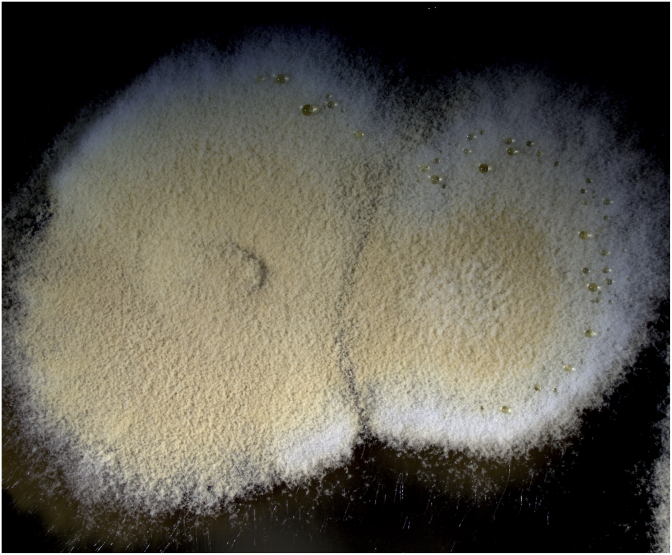
Close-up detail of a mature *Arthroderma uncinatum* colony showing active metabolic guttation.

**Fig. 4:**
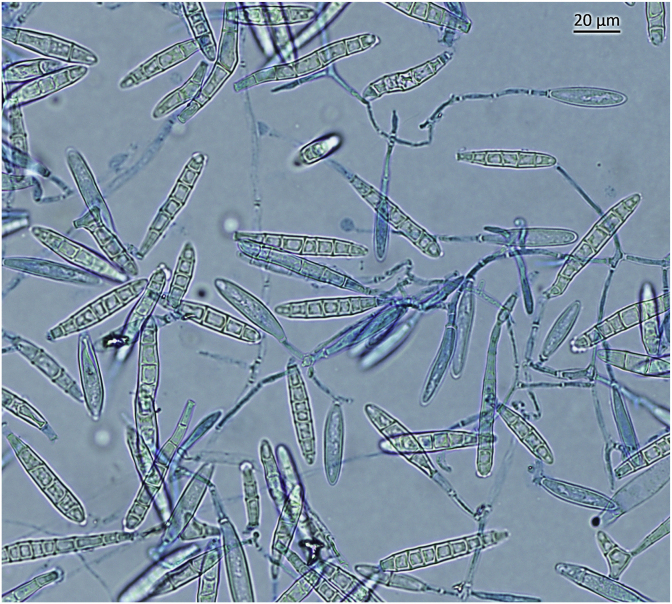
Micromorphological characteristics of *Arthroderma uncinatum* with lactophenol cotton blue (40x). Abundant macroconidia hyaline, smooth and thick walled, cigar-shaped divided into 5 to 9 internal cells and non-branched conidiophores.

After 7 days of incubation (Day +7), fungal growth was observed in all cultures. Importantly, cultures obtained independently from each of the two lesions—one on the right forelimb and one on the right hindlimb—were both positive for dermatophyte growth. In addition to these colonies, other fungal growth was also present. The dermatophyte colonies were macroscopically characterized by a whitish color and feathery margins. To further characterize these isolates, subcultures were established on MYC and SDA and incubated at room temperature to assess the production of pigments and reproductive structures. By Day 21, the colonies macroscopically demonstrated rapid growth, presenting as flat, dense, and conspicuously granular-to-powdery in texture (Fig. 2). The obverse surface was characterized by a distinct cream-to-tan or pale buff coloration at the center, surrounded by a prominent, white downy-to-floccose peripheral zone with irregular, radiating borders (Fig. 2a–c). Notably, mature colonies exhibited active metabolic guttation, with the accumulation of small, clear exudate droplets across the granular surface matrix (Fig. 3). Examination of the reverse side on both the SDA and MYC (Fig. 2b–d) media revealed an intense, deep dark-brownish to black central pigmentation that gradually diffused into a warm golden-yellow or amber hue towards the advancing periphery. Micromorphological analysis revealed abundant macroconidia arising directly from septate vegetative hyphae, borne either terminally or laterally on short, non-branched conidiophores. The macroconidia occasionally displayed a slight apical tapering or blunt distal ends, and were predominantly elongated, spindle-to cigar-shaped, and smooth; they possessed distinctly thickened double cell walls, with multiple regular transverse septations, consistently dividing each individual conidium into 5 to 9 distinct internal cells. Conversely, microconidia were sparse, small, and inconsistently distributed throughout the examined fields, confirming a highly specialized macroconidial-dominant phenotype (Fig. 4).

### 2.3 Molecular identification and sequencing

Genomic DNA was extracted from young mycelia harvested from pure cultures using the commercial DNeasy Blood & Tissue Kit (QIAGEN, Hilden, Germany), strictly adhering to the manufacturer's instructions for filamentous fungi. Molecular identification was achieved by targeting the nuclear ribosomal ITS region. Polymerase chain reaction amplification was performed using the universal fungal primer pair ITS1 (5′-TCCGTAGGTGAACCTGCGG-3′) and ITS4 (5′-TCCTCCGCTTATTGATATGC-3′), following previously established protocols [15]. The resulting PCR products were enzymatically purified to remove residual primers and dNTPs, and subsequently subjected to bidirectional automated Sanger sequencing. The raw forward and reverse electropherograms were chromatographically evaluated, trimmed, and assembled into a consensus sequence using AliView software (version 1.28). To establish identity, the consensus sequence was compared against reference nucleotide datasets available in the National Center for Biotechnology Information (NCBI) GenBank and the MYCOBANK databases using the Basic Local Alignment Search Tool (BLASTn); this comparison demonstrated a 99% query coverage, a non-significant E-value of 0.0, and a 100% sequence identity match with validated reference strains of *A. uncinatum* (syn. *T. ajelloi*).

The resulting consensus nucleotide sequence was deposited in the GenBank database and assigned the accession number PZ567627.

### 2.4 Therapeutic intervention and clinical follow-up

**Fig. 5:**
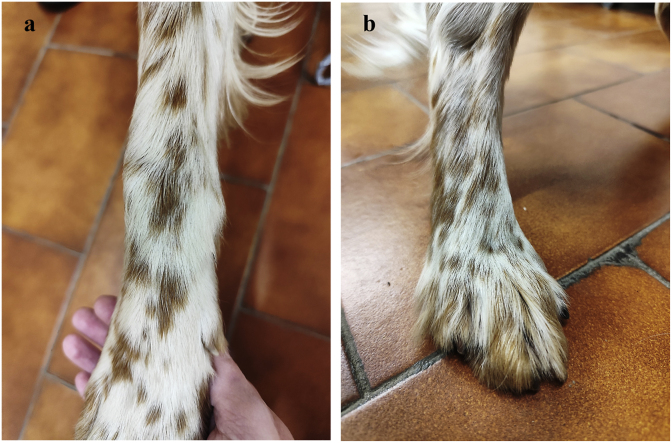
Complete clinical resolution of the dermatological lesions two weeks post-treatment: (**a**) metatarsal region; (**b**) metacarpal region.

Following the initial consultation (Day 0), while awaiting the culture results, the patient was started on a daily basis on a topical antiseptic (Betadine®, povidone-iodine), 1:10 dilutions. Following the macroscopic and microscopic identification of a dermatophyte (Day 21), topical treatment with a 1% clotrimazole cream (Canesten®, Bayer) was started, applying a thin layer on the affected areas twice daily for 3 weeks. While on the Canesten treatment, a definitive diagnosis of *A. uncinatum* was confirmed molecularly. After 3 weeks of treatment (Day +42), the patient was rechecked and, given the complete resolution of the clinical lesions, the topical treatment was discontinued. A further follow-up took place 2 weeks later (Day +56), during which no flares were observed, alongside the *ad integrum* restitution of the hair coat (Fig. 5). To confirm a mycological cure, a dermatophyte culture with a hair pluck technique was performed. No growth was observed 21 days post-incubation (Day +77).

## 3 Discussion

The isolation of *A. uncinatum* from active cutaneous lesions in a dog represents an exceptional clinical event, as this geophilic fungus is traditionally classified as a soil saprophyte with negligible pathogenic potential [9,10]. While *T. ajelloi* possesses documented keratinolytic activity that allows it to thrive on organic debris in environmental substrates, its adaptation to animal hosts is minimal [6,7]. In veterinary medicine, the organism is predominantly recovered as a transient, non-pathogenic contaminant of the hair coat in healthy pets [13]. Distinguishing true tissue invasion from incidental environmental contamination is therefore essential.

In the present case, the pathogenic role of *A. uncinatum* is supported by three concurrent findings: (i) the absence of other dermatological pathogens, (ii) the presence of cutaneous lesions consistent with dermatophytosis, and (iii) the complete and rapid clinical, as well as mycological, remission achieved *ex juvantibus* solely through targeted topical antifungal therapy. Collectively, these findings strongly support a genuine infectious process rather than incidental environmental carriage or contamination.

The physical presentation of the lesions observed in this patient underscores the significant clinical polymorphism that characterizes atypical dermatophytosis (e.g., a more inflamed and exudative metacarpal lesion vs a less inflamed and dry metatarsal lesion). This clinical heterogeneity, combined with the distal localization on the extremities, frequently leads to diagnostic delays due to symptomatic overlaps with bacterial pyoderma, parasitic infestations, traumatic injuries, or immune-mediated disorders [5].

The epidemiological context of this case aligns with established ecological frameworks for geophilic dermatophytosis [7]. The patient was a young hunting dog housed outdoors with access to a garden. The animal's routine outdoor training involved prolonged, direct contact with soil and dense vegetation, which significantly increased the probability of the mechanical inoculation of fungal propagules into the dermis. Furthermore, the environmental persistence of such geophilic agents is favored by the warm, humid climate characteristic of Southern Italy [2]. It is highly probable that minor, unnoted micro-trauma that occurred during hunting training disrupted the cutaneous barrier, facilitating the saprophytic-pathogenic transition of the fungal macroconidia into the stratum corneum.

Interestingly, the older cohabiting dog remained completely asymptomatic despite living in the same environment. Although host-related factors may have contributed to susceptibility, the differing clinical outcome is likely attributable to the younger dog's greater level of outdoor hunting training activity, which may have increased its exposure to microhabitats rich in keratinous debris and opportunistic geophilic fungi [2,3].

From a diagnostic perspective, the initial trichogram did not show the ectothrix or endothrix parasitism classically present in dermatophytosis, as documented in infections caused by poorly adapted geophilic or sylvatic strains [16]. This diagnostic ambiguity reinforces the necessity of combining phenotypic and genotypic approaches in mycology [14].

Macroscopic characterization on SDA and MYC provided critical preliminary clues, notably the rapid development of granular, cream-tan colonies exhibiting a deep dark-brown to black reverse pigmentation. The observation of active metabolic guttation across the mature colony surface further highlighted the active metabolic state of the isolate. Micromorphologically, the predominance of large, smooth, thick-walled, multi-septate (5–12 cells) cigar-shaped macroconidia, coupled with sparse microconidia, strongly pointed towards *A. uncinatum* according to standard mycological keys [11]. However, given the increasing taxonomic revisions and the morphological overlap among related soil-associated dermatophytes, definitive identification required molecular confirmation [14]. Sequencing of the ITS region of ribosomal DNA has emerged as the gold standard for resolving atypical or polymorphic dermatophyte phenotypes [9]. In our report, the consensus sequence demonstrated 100% nucleotide identity with validated reference strains of *A. uncinatum*.

From a public health perspective, the confirmation of *A. uncinatum* as a primary animal pathogen is of importance as it might act as opportunistic agents in humans [17]. Zoonotic transmission may occur through direct exposure to contaminated animal dander, hair, or soil-laden fomites within shared domestic microhabitats [9], which could trigger pronounced inflammatory cutaneous reactions escaping standard diagnostic algorithms [12]. Consequently, documenting canine cases is vital not only for veterinary surveillance but also for identifying potential bio-reservoirs that pose infectious risks to pet owners, hunters, and veterinary practitioners [18].

Fortunately, despite its inflammatory clinical appearance, the infection proved highly responsive to conservative therapeutic measures [1]. The application of a topical 1% clotrimazole cream twice daily for 3 weeks resulted in complete clinical resolution without the need for systemic antifungal administration. The formal follow-up evaluation at Day +42 confirmed full hair regrowth (*ad integrum* restitution) and achieved a completely negative mycological status (Day +77), demonstrating that localized geophilic infections can be successfully managed with targeted, non-invasive topical protocols.

This case report documents a molecularly confirmed case of canine dermatophytosis caused by *A. uncinatum* presenting as atypical, inflammatory distal limb lesions. The clinical course underscores the importance of including rare geophilic fungi in the differential diagnosis of focal alopecic lesions, particularly in outdoor working breeds exposed to specific environmental microhabitats. This case demonstrates the importance of a mycological examination, always including a culture. While conventional phenotypic identification remains a cornerstone of diagnostic mycology, ITS amplification and sequencing is an indispensable tool for achieving a definitive taxonomic diagnosis when dealing with rare, opportunistic, or morphologically atypical pathogens. Within a comprehensive "One Health" approach, monitoring these rare geophilic infections provides critical epidemiological insights into the shifting dynamics of opportunistic fungal pathogens across shared human and animal environments.

## Ethical form

No funding has been provided for this work.

## CRediT authorship contribution statement

**Rossella Samarelli:** Conceptualization, Data curation, Formal analysis, Investigation, Methodology, Validation, Visualization, Writing – original draft, Writing – review & editing. **Domenico Otranto:** Conceptualization, Funding acquisition, Resources, Supervision, Validation, Writing – review & editing. **Mara Miglianti:** Investigation, Methodology. **Marcos Antonio Bezerra-Santos:** Methodology, Writing – review & editing. **Gustavo Giusiano:** Conceptualization, Data curation, Investigation, Methodology, Project administration, Supervision, Validation, Writing – review & editing.

## Conflict of interest

There are none.
